# A shift from exploitation to interference competition with increasing density affects population and community dynamics

**DOI:** 10.1002/ece3.2284

**Published:** 2016-07-01

**Authors:** Erica M. Holdridge, Catalina Cuellar‐Gempeler, Casey P. terHorst

**Affiliations:** ^1^ Department of BiologyCalifornia State University, Northridge18111 Nordhoff StreetNorthridgeCalifornia91330; ^2^ Department of Ecology & Evolutionary BiologyYale University165 Prospect StreetNew HavenConnecticut06511; ^3^ College of Natural SciencesUniversity of Texas at Austin120 Inner Campus DriveAustinTexas78712

**Keywords:** Bacteria, density dependence, frequency‐dependent selection, intraspecific competition, microcosm, protozoa

## Abstract

Intraspecific competition influences population and community dynamics and occurs via two mechanisms. Exploitative competition is an indirect effect that occurs through use of a shared resource and depends on resource availability. Interference competition occurs by obstructing access to a resource and may not depend on resource availability. Our study tested whether the strength of interference competition changes with protozoa population density. We grew experimental microcosms of protozoa and bacteria under different combinations of protozoan density and basal resource availability. We then solved a dynamic predator–prey model for parameters of the functional response using population growth rates measured in our experiment. As population density increased, competition shifted from exploitation to interference, and competition was less dependent on resource levels. Surprisingly, the effect of resources was weakest when competition was the most intense. We found that at low population densities, competition was largely exploitative and resource availability had a large effect on population growth rates, but the effect of resources was much weaker at high densities. This shift in competitive mechanism could have implications for interspecific competition, trophic interactions, community diversity, and natural selection. We also tested whether this shift in the mechanism of competition with protozoa density affected the structure of the bacterial prey community. We found that both resources and protozoa density affected the structure of the bacterial prey community, suggesting that competitive mechanism may also affect trophic interactions.

## Introduction

Intraspecific competition is a major factor driving population dynamics (Schoener 1973), often outweighing the effects of interspecific competition (Connell 1983; de Villemereuil and López‐Sepulcre 2011). As population density increases, population growth rates should decrease as resource availability per individual diminishes and intraspecific competition increases. An increase in resources or productivity can alleviate the negative effects of competition (McAllister et al. 1972). For example, aboveground competition in a native perennial grass, *Schizachyrium scoparium*, was most intense where light was limiting and decreased significantly as light availability increased (Wilson and Tilman 1993). In the same system, belowground competition was most intense in plots with limited nitrogen.

However, the relationship between the strength of competition and resource availability is not always so simple. The paradox of enrichment predicts that increasing resources could result in extinction in simple predator–prey systems (Huffaker et al. 1963; Rosenzweig 1971). In a temperate herb population, *Chenopodium album*, competition was most intense when both light availability and nitrogen availability were highest, but was less intense when one or both of these resources were limiting (Nicotra and Rodenhouse 1995). Plant biomass was highest in environments that produced the most intense competition (Nicotra and Rodenhouse 1995). This counterintuitive effect of increased resources could occur because some other resource becomes limiting as biomass increases (e.g., Harpole and Suding 2011). Another possibility is that resource availability shifts the mechanism of competition.

Competition can occur through either exploitative or interference mechanisms. Exploitative competition is an indirect negative effect of individuals on each other that occurs through use of a shared resource and has been considered heavily in ecological theory (MacArthur and Levins 1967; MacArthur 1972; Simberloff 1982; Tilman 1990; Holt and Polis 1997). Interference competition is a direct form of competition that occurs when individuals inhibit the ability of others to access a shared resource, either aggressively (hoarding, guarding, allelopathy, etc.) or passively (bumping, unintentional blocking, etc.). While the strength of exploitative competition depends on resource availability, the strength of interference competition may not depend on resource availability (Arditi and Ginzburg 1989). Models of exploitative competition assume that consumers encounter resources randomly at a rate that is proportional to resource density (Arditi and Ginzburg 1989); an increase in resources will result in an increase in the encounter rate. For example, imagine a population of squirrels that compete for nuts exploitatively, in that each nut eaten by a squirrel reduces the number of nuts available for other squirrels. In that case, squirrel fitness highly depends on the number of nuts. However, interference competition becomes more important when consumer behavior affects the encounter rate. In the case of many squirrel species, these behaviors include territoriality, where individuals guard and defend highly productive trees, and hoarding (Gordon 1936). If one large squirrel hoards most of the nuts, adding more nuts will provide little benefit to other squirrels because it will result in more hoarding by the large squirrel. As interference competition increases, the relative importance of resources may decrease (Arditi and Ginzburg 1989).

In addition to their effects on population dynamics, alternate mechanisms of intraspecific competition can have community‐level effects. For example, apparent competition is an indirect effect that can occur through a shared predator and can have important consequences for the diversity of prey communities (Holt 1977). Yet, if predator populations are limited by some factor other than the availability of their prey resource, such as intraspecific interference competition, this can have cascading effects on the prey community (Holt 1977). Regardless of the mechanism of change in trophic interactions, we expect the mechanism of competition to have consequences for the diversity of the prey community.

One fundamental question that remains unanswered is whether the relative strength of interference competition remains fixed for a given population at a specific time and location or whether it varies with population density. In other words, we do not know whether interference competition is “characteristic” or “shifting” (DeLong and Vasseur 2011). There is little consensus in the literature about the nature of the relationship between the relative strength of interference competition and consumer density. Fussmann et al. (2005) found that the effects of interference competition on consumer functional response were only significant at unusually high consumer densities. In contrast, Kratina et al. (2009) found interference competition to have a significant effect on consumer functional response at a wide range of consumer densities, including low densities. Delong and Vasseur (2013) showed that the strength of exploitative and interference competition had an inverse relationship in populations of *Didinium* preying upon *Paramecium* but do not propose any mechanism as to why this may be. To test how the relative strengths of interference and exploitation competition change with consumer density, we manipulated resource levels and population density of a single protozoan species, *Colpidium* sp., in laboratory microcosms to evaluate their effects on population dynamics. We solved a dynamic predator–prey model for parameters of the functional response using population growth rates measured in our experiment to determine whether the mechanism of competition shifted between exploitative and interference competition and whether any shift that did occur affected the bacterial prey community.

## Methods

### Protozoa population dynamics

We isolated one species of ciliated protozoan, *Colpidium* sp., from purple pitcher plants (*Sarracenia purpurea*) in the Apalachicola National Forest in northern Florida. Several ciliate morphotypes have been identified in this system, and genetic sequencing confirmed that different morphotypes are different species and individuals of the same morphotype belong to the same species (terHorst 2011). We used a culture isolated from a single leaf to inoculate laboratory microcosms in sterile 50‐mL macrocentrifuge tubes with 25 mL of sterile water and 5 mL of bacterial stock culture. The bacteria were collected from several pitcher plants. We added either 3 or 12 mg of freeze‐dried bloodworms as a basal resource to each microcosm and assume that the density of bacteria scales proportionally with the density of basal resources. We quantified the density of *Colpidium* in the stock culture by thoroughly mixing the culture then fixed 1 mL of sample using Lugol's iodine. We gently centrifuged (1 min at 300 rpm) the sample to concentrate cells at the bottom of each tube and sampled 0.1 mL from the bottom of the tube, which was placed on a Palmer counting cell and counted under light microscopy. This method, as opposed to counting 0.1 mL of sample directly from the culture, enabled us to increase our sampling effort 10‐fold and better ensure that the subsamples represented the microcosm densities as a whole. We factorially manipulated five initial density treatments – approximately 0, 26, 77, 132, and 263 cells/mL based on the estimated density of the stock culture – at each resource level, for a total of ten treatments and 100 microcosms (*n* = 10).

Microcosms were allowed to grow at ambient laboratory temperature (approximately 25°C) for 72 h. After this period of growth, we mixed each microcosm and fixed a 1‐mL sample using Lugol's iodine. We quantified final cell densities using a Palmer counting cell under light microscopy, as described for the stock culture above. Per capita growth rates were calculated based on the initial and final cell densities by dividing the difference between final and initial cell densities by the initial cell density. We examined the effects of resource level, initial protist density, and their interaction on per capita growth rates using a generalized linear model with a Gaussian distribution. A Gaussian error distribution produced lower Akaike information criterion (AIC) values than any other error distribution we tested.

We calculated the intrinsic growth rate *r* as

(1) $$r=\frac{\ln(N_{f}/N_{i})}{\Delta t}$$

where *N*_i_ and *N*_f_ are the initial final protist densities, respectively, and Δ*t* is the time elapsed between the initial and final measurements. We then used *r* to calculate the rate of change of the consumer population d*C/*d*t* as

(2) $$\frac{dC}{dt}=rN_{i}$$

which we later used in our consumer–resource model.

We estimated consumer foraging rates using resource and consumer density according to the Hassell–Varley–Holling trophic function (Sutherland 1983; Delong and Vasseur 2013; eq. (3)):

(3) $$f\left(R,C\right)=\frac{aRC^{m}}{1+ahRC^{m}}$$

where *a* is the attack rate, *R* is the resource availability, *C* is the consumer density, *h* is the handling time, and *m* describes the intensity of interference in the system. The interference parameter *m* has two special cases. *m *=* *0 describes a completely exploitative system where foraging rates are solely determined by resource availability. In contrast, *m *=* *−1 indicates a system that is completely interference‐based and foraging rates are determined by the ratio of resource to consumer density. The Hassell–Varley–Holling trophic function is the most widely used functional response that includes a term for interference competition (see Appendix S1 for more detail on the behavior of this function). Two other functions, the Beddington–DeAngelis (Beddington 1975; DeAngelis et al. 1975) and the Crowley–Martin (Crowley and Martin 1989), are also used, although less frequently, when considering the effects of interference competition on feeding rates. The Beddington–DeAngelis function produces results that are not distinguishable from those of the Hassell–Varley–Holling function when fit to empirical data, so there is no reason to believe that using the Beddington–DeAngelis function would affect the results of our study (Skalski and Gilliam 2001). Fits produced by the Crowley–Martin function do differ from those of the Hassell–Varley–Holling function (Skalski and Gilliam 2001). However, this function assumes that the effect of interference on feeding rates remains the same under all resource levels. For this reason, the Crowley–Martin function is not appropriate for our data set.

We used a MacArthur–Rosenzweig predator–prey model (Rosenzweig and MacArthur 1963) and equation (3) to produce the following equation for consumer population growth rates (Delong and Vasseur 2013):

(4) $$\frac{dC}{dt}=C\left(ef\left(R,C\right)-\mu \right)$$

where *e* is the conversion efficiency and *μ* is the natural mortality rate. Using the calculated rate of change for the consumer populations (d*C/*d*t*), resource availability, and consumer density for each microcosm, we were able to simultaneously estimate the attack rate (*a*), handling time (*h*), and intensity of interference competition (*m*) in each microcosm (sensu “Method 3” DeLong and Vasseur 2011) while holding conversion efficiency (*e *=* *0.8) and natural mortality (*μ *= 0.1) constant using maximum‐likelihood models in the R package “bbmle” (Bolker and R Development Core Team 2014). We used generalized linear models to determine whether the intensity of interference competition, m, changes with population density. We also used AIC for model comparison to determine whether the dynamics between growth rates and interference intensity can be explained by resource availability. When ΔAIC > 2, we considered models significantly different from one another (Richards 2005).

### Bacterial community dynamics

At the end of the experiment, we sampled bacteria from five randomly chosen microcosms from each of the ten treatments by filtering water from each microcosm through a 0.22‐μm pore filter. The filter was preserved in ATL buffer and frozen at −80°C until further sample processing. Genomic DNA was extracted from the filters with Blood and Tissue DNA extraction kits (Qiagen, Hilden, Germany) with two modifications to the protocol. First, samples were lysed by vortexing at maximum speed for 1 min with 0.5‐mL sterile microbeads. Second, samples were incubated at 57°C for 15 min with proteinase K for chemical lysis. All subsequent steps were performed as indicated by the kit. Sequencing of the V4 region of the 16S rRNA gene (Wang and Qian 2009) was carried out at the Genomic Sequencing Analysis Facility at the University of Texas at Austin. Sequences were quality‐filtered and processed using QIIME (v. 1.8.0, Caporaso et al. 2010) and clustered using UCLUST with a 97% similarity cutoff (Edgar 2010) into operational taxonomic units (OTUs) based on the GreenGenes database (DeSantis et al. 2006). The effects of protozoa density and resource level on bacterial richness and evenness were evaluated with two‐way ANOVAs. We used a redundancy analysis (RDA) to reduce OTU composition to the first two RDA axes and tested the effects of protozoa density and resource level using (perMANOVA) on RDA scores.

## Results

**Figure 1:**
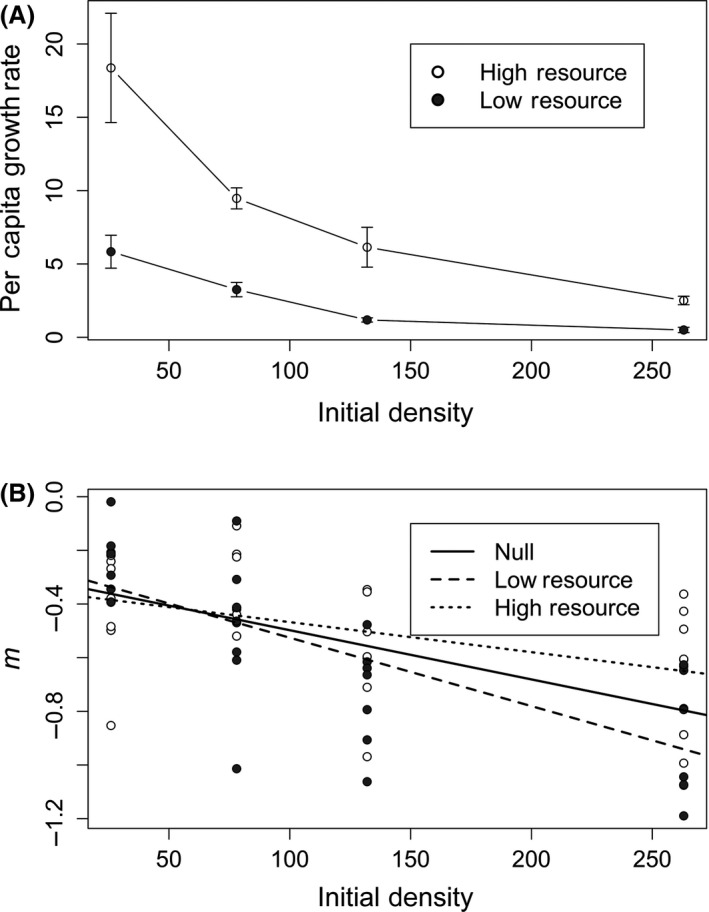
(A) Per capita growth rates of Colpidium in response to initial density (cells/mL) and resource availability (low = 3 mg; high = 12 mg). (B) Estimates of the mutual interference m parameter in response to initial density. Two fit models are shown, a null model (solid line; adjusted *R* ^2^ = 0.28), which includes only initial density, and a saturated model (dashed lines; adjusted *R* ^2^ = 0.27), which includes both initial density and resource availability. The null model provided the best fit and shows a significant effect of initial density (*t* = −4.92, *P* < 0.001) on *m*.

We removed 23 samples from our analyses because they were cross‐contaminated during the course of the experiment, bringing the total number of samples to 77 (Appendix S2; Table S2). Per capita growth rates of *Colpidium* decreased significantly with increasing initial density in both low‐ and high‐resource treatments (Table 1; Fig. 1A). The high‐resource treatment always had higher growth rates than the low‐resource treatment (Table 1; Fig. 1A). However, there was a significant interaction between resource availability and initial density (Table 1), such that the difference between per capita growth rates in low‐ and high‐resource treatments was greatest at low densities. At high initial densities, resource treatment had relatively little effect on per capita growth rates (Fig. 1A).

**Table 1:** Summary table of final generalized linear model, ANOVA and perMANOVA results. Bolded values indicate significant effects

Response and explanatory variables	*F*	df	*P*
*Colpidium* per capita growth rates			
Resource	31.95	1	**<0.001**
Density	39.20	1	**<0.001**
Resource × density	9.13	1	**0.004**
Estimated *m*			
Density	85.33	1	**<0.001**
Bacterial richness			
Resource	9.78	1	**0.003**
Density	0.01	1	0.922
Resource × density	0.12	1	0.736
Bacterial evenness			
Resource	9.45	1	**0.004**
Density	0.01	1	0.946
Resource × density	0.16	1	0.692
Bacterial composition			
Resource	26.52	1	**0.001**
Density	12.36	1	**0.001**
Resource × density	1.73	1	0.189

There was a significant decrease in the interference parameter (*m*), corresponding to a shift from exploitative to interference competition, as density increased (Table 1; Fig. 1B). As density increased, *m* shifted from −0.24 on average (and thus more dominated by exploitation) to −1.03 on average (and thus more dominated by interference). We also used model comparison to evaluate a null model, which only included initial density as a predictor, and a more saturated model that included both initial density and resource availability (Fig. 1B). The AIC value for the null model was −5.6, while the AIC value for the saturated model was −5.5 (ΔAIC 0.1), indicating that the two models are indistinguishable from one another. In addition, neither resource availability (*P* = 0.89) nor the interaction between initial density and resource availability (*P* = 0.22) had significant effects on the *m* interference parameter. We also explored model behavior for different values of e and *μ*. Reasonable model fits could not be found for low values of conversion efficiency, because whenever fits were significant, handling time was negative. This is likely a result of naturally high conversion efficiencies in *Colpidium*. Lower values of natural mortality resulted in no qualitative changes to our results, except the saturated model showed a significant interaction between population density and resource availability.

**Figure 2:**
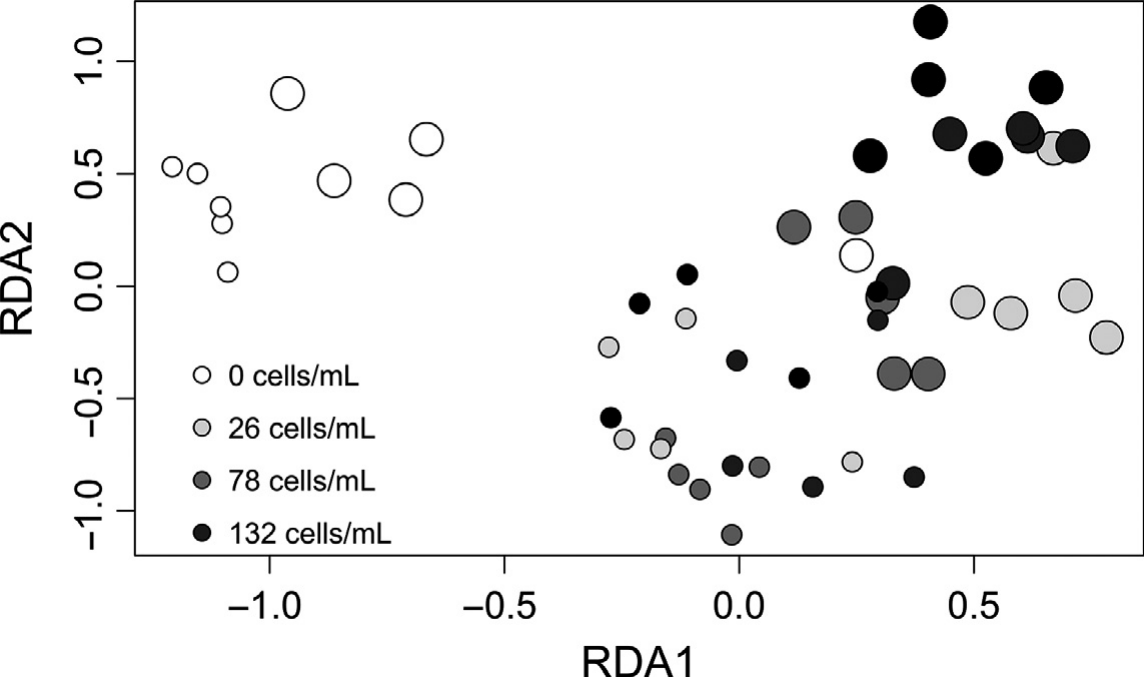
Ordination plot of bacterial community composition based on the first two axes of site scores from a redundancy analysis. Each point represents one replicate microcosm. Grayscale shading indicates initial protists density (cells/mL) and symbol size represents resource availability (low [large circles] = 3 mg; high [small circles] = 12 mg).

**Figure 3:**
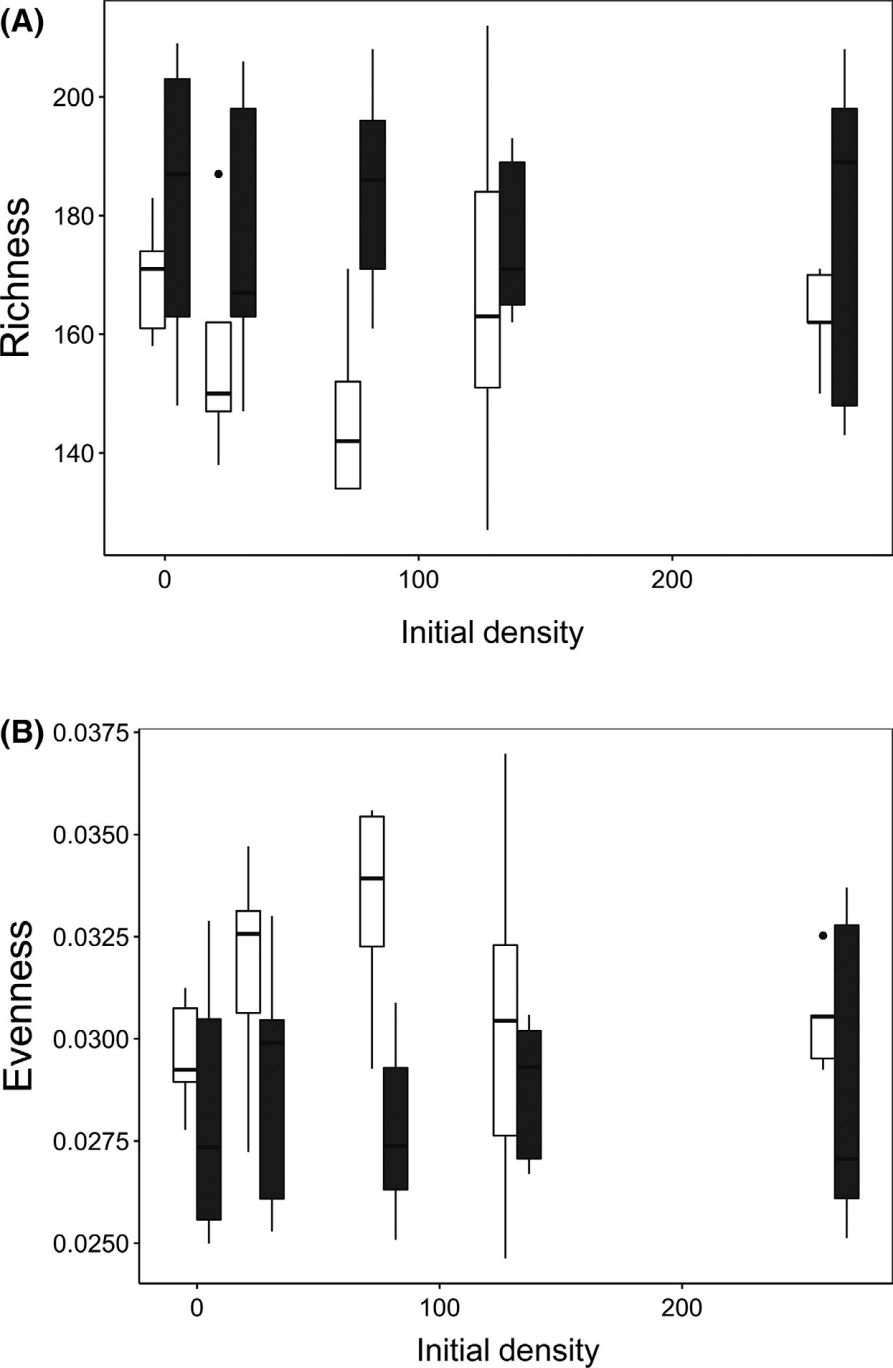
Effects of resource availability (low [filled] = 3 mg; high [open] =12 mg) and protist initial density (cells/mL) on bacterial community species (A) richness and (B) evenness.

Resource availability and *Colpidium* density had a significant effect on bacterial community composition (Table 1; Fig. 2). Bacterial communities from the high‐resource treatment had significantly lower species richness (Table 1; Fig. 3A) and significantly greater evenness (Table 1; Fig. 3B) than those from the low‐resource treatment. However, *Colpidium* density did not have a significant effect on either species richness (Table 1; Fig. 3A) or evenness (Table 1; Fig. 3B).

## Discussion

As we expected, population growth rates decreased with decreasing resources and increasing population densities, suggesting that *Colpidium* populations experience resource limitation and intraspecific competition. Consequently, we expected resource levels to be more important at higher densities, where competition was likely strongest. Instead, we observe a subadditive effect in which the effects of resource levels decreased with increasing density. As a result, the growth rates of dense populations seemed to be relatively unaffected by resource availability. One reason for this surprising result could be a shift from resource‐dependent exploitative competition to interference competition that was independent of resource level.

Interference competition can reduce resource use independent of resource availability (Arditi and Ginzburg 1989). The intensity of interference competition was estimated here as the *m* parameter in equation (2), where more negative values indicate a population that is more influenced by interference competition. Our microcosm populations showed a significant decrease in *m* with increasing density, indicating that competition shifts from exploitation to interference with increasing density. This shift in *m* was best explained by protist density and not resource levels, suggesting that the shift in *m* was due to an increase in interference competition rather than a decrease in exploitative (resource‐dependent) competition.

Mechanisms of interference competition between ciliates are not well documented. Interference competition in *Colpidium* populations likely occurs because individuals are blocking access to resources through passive mechanisms. As microcosms become crowded, it is common to observe individuals bumping into one another as they search for food. Collisions, which increase search time, have been documented as one mechanism of interference competition that occurs between ciliates as well as other protists (Habte and Alexander 1978; Fox 2002). We have also observed populations of ciliates, including *Colpidium*, forming mats on the surface of any type of structure in the microcosm, presumably because bacteria aggregate there. This behavior could block additional consumers from accessing these aggregations of bacteria. This is similar to many other organisms, both sessile and motile, that compete for access to ideal habitat in a spatially heterogeneous environment. For example, barnacles as well as many other sessile marine species compete for limited space on ideal settlement substrata and turtles as well as other ectotherms compete for limited basking areas (Schoener (1983) refers to this as *preemptive competition*). These same processes could occur in other systems, through either passive or aggressive modes of interference. For example, when populations were dense and prey were unevenly distributed in the environment, *Callinectes sapidus* (blue crabs) blocked access to prey, resulting in interference competition that decreased their foraging success (Clark et al. 1999). Over longer time periods, this could reduce population growth rates, as individuals spend more energy competing with conspecifics and less on reproduction (Schoener 1973).

Both resource level and protist density affected the composition of the bacterial community. Communities from the same resource and protist density treatments tended to cluster together in terms of community composition (Fig. 2). We cannot discern whether these differences are the direct result of predator density (Leibold 1996) or the indirect effect of predator density on competitive interactions among bacteria. However, we know from Holt (1977) that the diversity of prey communities is expected to depend on whether predator populations are regulated by resource availability or by some other factor, such as interference competition. This expectation is consistent with our results. We show here that the intensity of interference competition increased with protist density, so it is likely that some portion of the variation in bacterial community composition was due to this shift in competition mechanisms. Traits that confer an advantage to protists experiencing interference competition can also have consequences for the composition of the bacterial community. For example, larger bodied protists are known to be superior competitors (Kneitel 2012). Many gape‐limited protists, like *Colpidium*, show size specificity in the kinds of bacteria they consume (Fenchel 1980). If larger bodied individuals access resources and feed more readily in population because of their competitive advantage, this could lead to a shift in the bacterial community as a result of size‐specific feeding. Further research on the relative importance of predator density and competitive mechanisms on prey community composition would shed light on the patterns driving species diversity in communities.

In our experiment and model, we manipulated basal resources and assumed that this resulted in increased bacterial resources for *Colpidium*, although we did not quantify bacterial abundance. We believe this is a reasonable assumption because most natural systems show a proportional increase in abundance at each trophic level in response to bottom‐up effects (McQueen et al. 1986). Bacteria, in particular, typically increase in abundance proportionally with the amount of organic matter available in the environment (Billen et al. 1990) and this is specifically true of pitcher plant inquiline communities (Miller and terHorst 2012). Our models could be improved by directly including bacterial abundance, but we do not expect that this would qualitatively change our results.

One possible explanation for the differences in *Colpidium* growth rates between our two resource availability treatments is that resource level might have changed the relative proportion of edible bacteria in the community. The identity of individuals within a heterogeneous resource community like the one used in our experiment can influence the abundance and growth rates of the consumer. For example, Steiner (2001) studied a three trophic level food chain of basal nutrients, algae, and zooplankton under two levels of enrichment. The algal trophic level was composed either entirely of edible algae or a mixture of edible and inedible algae. Steiner found that the relative proportion of large inedible algae in the community increased with enrichment, which reduced the strength of top‐down control that zooplankton imposed on the algal community. As our experiment also used a heterogeneous resource community, it is possible that our resource availability treatments produced changes in the relative abundance of edible bacteria in the community, which could in part explain the observed changes in *Colpidium* growth rates. Similarly, changes in the nutritional quality of bacteria within each resource availability treatment could affect *Colpidium* population dynamics. Future work should address whether changes in bacterial community composition associated with changes in resources (Fig. 2) have consequences for *Colpidium* population growth rates.

We sampled the final densities of *Colpidium* populations 72 h after initiating the microcosms. However, laboratory populations of ciliates typically do not reach a stable population equilibrium. Rather, they exhibit transient population dynamics, in which populations grow, overshoot their carrying capacity, and decline. Transient dynamics are common in natural populations, as most populations live in variable environments and experience disturbances that perturb them away from their equilibrium densities. Consequently, transient dynamics are important in understanding the mechanisms that allow for persistence and coexistence (Hastings 2001, 2004). Mechanistic studies of populations that exhibit transient dynamics, like the one presented here, will lead to a better understanding of the ecological forces that shape the dynamics of natural populations.

An interesting potential implication of our study is that in addition to its effect on population dynamics, intraspecific competition can alter evolutionary trajectories through negative frequency‐dependent selection. For example, in Hawk–Dove game theory models (Maynard Smith and Price 1973; Maynard Smith 1982), individuals employ different behavioral strategies to compete for a shared resource. Rare phenotypes have the highest fitness when they are in low frequency and become less fit as their frequency increases. This mechanism of frequency‐dependent selection has been used to explain a number of evolutionary phenomena such as the maintenance of genetic diversity (Cockerham et al. 1972) and sympatric speciation (Doebeli 1996). If the mechanism of competition is driven by the prevalence of certain genotypes in the population, this could result in an eco‐evolutionary feedback, where the mechanism of competition, driven by population density, causes competitive traits to evolve, which would then affect population growth rates and density.

The topic of interference and exploitative competition, and functional responses in general, has received a great deal of attention from both empiricists and theoreticians. Fussmann (2008) provides an overview of the some of the challenges that can arise in experimental studies of functional responses. One challenge is to experimentally allow for mechanisms that can lead to consumer density dependence and use models that can capture these mechanisms (Skalski and Gilliam 2001). Previous studies have examined functional responses of consumers to a single prey species (Fussmann et al. 2005; Kratina et al. 2009). However, density dependence can occur in consumer populations when more than one prey species is present (Arditi and Ginzburg 1989). For example, prey‐switching behavior by a predator leads to a type III functional response (Holling 1959). By including multiple prey species in our experiment, we not only allowed for density‐dependent mechanisms, but also were able to explore community dynamics that emerge as a result of predation, direct competition, and apparent competition.

A second challenge is prey depletion, particularly in long‐term experiments. To account for this, previous studies have conducted experiments in chemostat environments (Fussmann et al. 2005; Kratina et al. 2009). While chemostats are a powerful tool for controlling nutrient levels and preventing resource depletion, such homogenous environments are rare in nature. For our experiment, we used 50‐mL macrocentrifuge tubes as microcosms, where the basal resources are heterogeneously distributed. Although this may lead to prey depletion in a long‐term experiment, our experiment was sufficiently short (72 h) to avoid this complication. In standard laboratory conditions (Lawler and Morin 1993) similar to our own, Orland (2003) found significant prey depletion in laboratory microcosms of *Colpidium striatum* occurred around 192 h. In addition, mechanisms leading to consumer density dependence can occur when resources are unevenly distributed in the environment (e.g., Clark et al. 1999). Conducting our experiment in an environment where resources are heterogeneously distributed allowed for this possible mechanism of consumer density dependence, again addressing the first challenge discussed.

Microbes in chemostats are often heralded as classic examples of exploitative competition (e.g., Arditi and Ginzburg 1989), but our results and those of other recent studies (e.g., Delong and Vasseur 2013) demonstrate that even simple microbial populations can be dominated by interference competition. Mutual interference competition is common across all taxa (DeLong and Vasseur 2011), but here we show that interference is a shifting parameter rather than a characteristic of a population. Intraspecific competition can also alter interspecific interactions by encouraging greater niche breadth, leading to niche overlap with more species (Vellend 2006). Understanding when and where to expect increases in the intensity of interference could provide a more mechanistic understanding of interspecific interactions. Further, intraspecific competition can increase food web stability by increasing predator diet diversity (Gross et al. 2009). Understanding mechanisms of competition could reveal layers of complexity about species and trophic interactions and evolution.

## Conflict of Interest

None declared.

## Supporting information


**Appendix S1.**
**Figure S1.** The Hassell–Varley–Holling trophic function plotted as a function of the interference parameter *m*.

**Appendix S2.**
**Table S2.** Cross‐contamination during the course of the experiment resulted in 23 samples being eliminated from our analyses. Shown here are the remaining 77 samples by treatment group.
