# Supplementary material for: A shift from exploitation to interference competition with increasing density affects population and community dynamics
Source: Ecol Evol. 2016 Jul 1;6(15):5333–41. doi: 10.1002/ece3.2284 (PMC4984507; doi:10.1002/ece3.2284)
Supplement: Supplementary file 1 — Appendix S1. Figure S1. The Hassell–Varley–Holling trophic function plotted as a function of the interference parameter m. [file ECE3-6-5333-s001.docx]

Appdendix S1


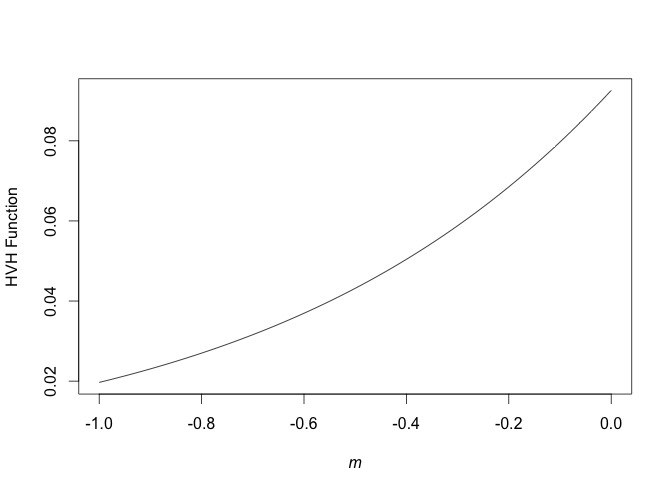


**Figure S1** The Hassell-Varley-Holling trophic function plotted as a function of the interference parameter *m* with an attack rate *a*=0.01, a handling time *h*=0.8, a consumer density of *C*=5, and a resource density of *R*=10. The shape of the curve remains the same for a wide range of parameter values.
