# Supplementary material for: A shift from exploitation to interference competition with increasing density affects population and community dynamics
Source: Ecol Evol. 2016 Jul 1;6(15):5333–41. doi: 10.1002/ece3.2284 (PMC4984507; doi:10.1002/ece3.2284)
Supplement: Supplementary file 2 — Appendix S2. Table S2. Cross‐contamination during the course of the experiment resulted in 23 samples being eliminated from our analyses. Shown here are the remaining 77 samples by treatment group. [file ECE3-6-5333-s002.docx]

**Appendix S2**

**Table S2.** Cross-contamination during the course of the experiment resulted in 23 samples being eliminated from our analyses. Shown here are the remaining 77 samples by treatment group.

|  | **Low Resources** | **High Resources** |
| --- | --- | --- |
| **0 cells/mL** | 8 | 8 |
| **26 cells/mL** | 8 | 8 |
| **77 cells/mL** | 8 | 8 |
| **132 cells/mL** | 7 | 6 |
| **263 cells/mL** | 8 | 8 |
